# Supplementary material for: SIMBA: Scalable Image Modeling using a Bayesian Approach, A Consistent Framework for Including Spatial Dependencies in fMRI Studies
Source: ArXiv. 2025 Nov 16:arXiv:2511.12825v1. Preprint. [Version 1] (PMC12754714)
Supplement: Supplement 1 [file NIHPP2511.12825v1-supplement-1.pdf]

Supplementary Material

This supplementary document includes details of the posterior distributions of the full set of parameters: $\boldsymbol{\Theta} = \{(\alpha_j)_{j=0}^J, (\boldsymbol{\theta}_\beta)_{j=0}^J, (\boldsymbol{\theta}_{\eta_i})_{i=1}^N, \sigma_\alpha^2, \sigma_\beta^2, \sigma_\eta^2, \sigma_\epsilon^2, a_\alpha, a_\beta, a_\eta, a_\epsilon\}$, that are used to draw samples from Gibbs sampling algorithm in Section 3.2, and the variational distributions with the ELBO function in Section 3.3. The full code are implemented in Pytorch and included in the GitHub repository https://github.com/y1zhong/apaper_simba.

Gibbs sampler

Given the reparameterized model in (3) and the specifications of the conjugate priors, we present the posterior distributions for each parameter below. Denote $\mathbf{1}_V \Phi = \widetilde{\Phi}$.
The full conditional distribution of each α_j, for $j = 0, \ldots, J$ is

$$\begin{aligned}
\alpha_j \mid \text{rest} &\sim N\left(\mu_{\alpha_j}, \nu_{\alpha_j}\right); \\
\nu_{\alpha_j} &= \left(\frac{\sum_{i=1}^N x_{ij}^2 \|\widetilde{\Phi}\|_F^2}{\sigma_\epsilon^2} + \frac{1}{\sigma_\alpha^2} \right)^{-1} \\
\mu_{\alpha_j} &= \nu_{\alpha_j} \cdot \frac{1}{\sigma_\epsilon^2} \sum_{i=1}^N x_{ij} (\widetilde{\mathbf{y}}_i - \mathbf{x}_i^\top \boldsymbol{\theta}_\beta - \boldsymbol{\theta}_{\eta_i} \Phi_\eta) \widetilde{\Phi}^\top.
\end{aligned}$$

The full conditional distribution of each $\boldsymbol{\theta}_{\beta_j}$ for $j = 0, \ldots, J$ is

$$\begin{aligned}
\boldsymbol{\theta}_{\beta_j} \mid \text{rest} &\sim N\left(\mu_{\beta_j}, \Sigma_{\beta_j}\right); \\
\Sigma_{\beta_j} &= \left(\frac{\sum_{i=1}^{N} x_{ij}^2}{\sigma_\epsilon^2} + \frac{1}{\sigma_\beta^2}\right)^{-1} I_L \\
\mu_{\beta_j} &= \Sigma_{\beta_j} \cdot \frac{1}{\sigma_\epsilon^2} \sum_{i=1}^{N} x_{ij} \left(\widetilde{\mathbf{y}}_i - \mathbf{x}_i^\top \boldsymbol{\alpha} \widetilde{\Phi} - \boldsymbol{\theta}_{\eta_i} \Phi_\eta\right)
\end{aligned}$$

The full conditional distribution of each $\boldsymbol{\theta}_{\eta_i}$ for $i = 1, \ldots, N$ is

$$\begin{aligned}
\boldsymbol{\theta}_{\eta_i} \mid \text{rest} &\sim N\left(\mu_{\eta_i}, \Sigma_{\eta_i}\right); \\
\Sigma_{\eta_i} &= \left(\frac{\Phi_\eta \Phi_\eta^\top}{\sigma_\epsilon^2} + \frac{1}{\sigma_\eta^2}\right)^{-1} \\
\mu_{\eta_i} &= \Sigma_{\eta_i} \left(\frac{1}{\sigma_\epsilon^2} \left(\widetilde{\mathbf{y}}_i - \mathbf{x}_i^\top \boldsymbol{\alpha} \widetilde{\Phi} - \mathbf{x}_i^\top \boldsymbol{\theta}_\beta\right)\right) \Phi_\eta^\top
\end{aligned}$$

The full conditional distribution of each variance parameter is

$$\sigma_\epsilon^2 \mid \text{rest} \sim \text{IG}\left(\frac{1 + NL}{2},\ \frac{1}{2}\|\widetilde{\mathbf{y}}_i - \mathbf{x}_i^\top \boldsymbol{\alpha} \widetilde{\Phi} - \mathbf{x}_i^\top \boldsymbol{\theta}_\beta - \boldsymbol{\theta}_{\eta_i} \Phi_\eta\|_2^2 + \frac{1}{a_\epsilon}\right), \quad a_\epsilon \mid \text{rest} \sim \text{IG}\left(1,\ \frac{1}{A^2} + \frac{1}{\sigma_\epsilon^2}\right)$$

$$\sigma_\eta^2 \mid \text{rest} \sim \text{IG}\left(\frac{1 + NL_\eta}{2},\ \frac{\sum_{i=1}^{N} \|\theta_{\eta_i}\|_2^2}{2} + \frac{1}{a_\eta}\right), \quad a_\eta \mid \text{rest} \sim \text{IG}\left(1,\ \frac{1}{A^2} + \frac{1}{\sigma_\eta^2}\right)$$

$$\sigma_\beta^2 \mid \text{rest} \sim \text{IG}\left(\frac{1 + (J+1)L}{2},\ \frac{\sum_{j=0}^{J} \|\theta_{\beta_j}\|_2^2}{2} + \frac{1}{a_\beta}\right) \quad a_\beta \mid \text{rest} \sim \text{IG}\left(1,\ \frac{1}{A^2} + \frac{1}{\sigma_\beta^2}\right)$$

$$\sigma_\alpha^2 \mid \text{rest} \sim \text{IG}\left(\frac{1 + J + 1}{2},\ \frac{\sum_{j=0}^{J} \alpha_j^2}{2} + \frac{1}{a_\alpha}\right), \quad a_\alpha \mid \text{rest} \sim \text{IG}\left(1,\ \frac{1}{A^2} + \frac{1}{\sigma_\alpha^2}\right)$$

Mathematical details in VI algorithm

Under the framework of mean field variational inference, we approximate the posterior distributions using

$$
\begin{aligned}
p(\boldsymbol{\Theta}|\mathbf{X},\mathbf{Y}) \approx q(\boldsymbol{\Theta}) &= \prod_{m=1}^{M} q(z_m) \\
&= \left(\prod_{j=0}^{J} q(\alpha_j) q(\boldsymbol{\theta}_{\beta_j})\right)\left(\prod_{i=1}^{N} q(\boldsymbol{\theta}_{\eta_i})\right) q(\sigma_\beta^2) q(\sigma_\eta^2) q(\sigma_\epsilon^2) q(a_\beta) q(a_\eta) q(a_\epsilon)
\end{aligned}
$$

For any m-th latent variable, the optimal $q(z_m)$ is given by:

$$
q(z_m) \propto \exp\left\{E_{-m}[\log p(z_m|z_{-m},\mathbf{X},\mathbf{Y})]\right\}.
$$

With derivation, we state the distributions under $q(\cdot)$ for each parameter as below:

$$
\begin{aligned}
\alpha_j \mid \text{rest} &\sim N\left(\mu_{\alpha_j}, \nu_{\alpha_j}\right); \\
\nu_{\alpha_j} &= \left(\frac{\sum_{i=1}^{N} x_{ij}^2 \|\widetilde{\Phi}\|_F^2}{E_q(\sigma_\epsilon^2)} + \frac{1}{E_q(\sigma_\alpha^2)}\right)^{-1} \\
\mu_{\alpha_j} &= \nu_{\alpha_j} \cdot \frac{1}{E_q(\sigma_\epsilon^2)} \sum_{i=1}^{N} x_{ij} (\widetilde{\mathbf{y}}_i - \mathbf{x}_i^\top E_q(\boldsymbol{\theta}_\beta) - E_q(\boldsymbol{\theta}_{\eta_i})\Phi_\eta)\widetilde{\Phi}^\top
\end{aligned}
$$

$$
\begin{aligned}
\boldsymbol{\theta}_{\beta_j} \mid \text{rest} &\sim N\left(\mu_{\beta_j}, \Sigma_{\beta_j}\right); \\
\Sigma_{\beta_j} &= \left(\frac{\sum_{i=1}^{N} x_{ij}^2}{E_q(\sigma_\epsilon^2)} + \frac{1}{E_q(\sigma_\beta^2)}\right)^{-1} I_L \\
\mu_{\beta_j} &= \Sigma_{\beta_j}\left(\frac{1}{E_q(\sigma_\epsilon^2)} \sum_{i=1}^{N} x_{ij} \left(\widetilde{\mathbf{y}}_i - \mathbf{x}_i^\top E_q(\boldsymbol{\alpha})\widetilde{\Phi} - E_q(\boldsymbol{\theta}_{\eta_i})\Phi_\eta\right)\right)
\end{aligned}
$$

$$\begin{aligned}
\boldsymbol{\theta}_{\eta_i} \mid \text{rest} &\sim N\left(\mu_{\eta_i}, \Sigma_{\eta_i}\right); \\
\Sigma_{\eta_i} &= \left(\frac{\Phi_\eta \Phi_\eta^\top}{E_q(\sigma_\epsilon^2)} + \frac{1}{E_q(\sigma_\eta^2)}\right)^{-1} \\
\mu_{\eta_i} &= \Sigma_{\eta_i}\left(\frac{1}{E_q(\sigma_\epsilon^2)}\left(\widetilde{\mathbf{y}}_i - \mathbf{x}_i^\top E_q(\boldsymbol{\alpha})\widetilde{\Phi} - \mathbf{x}_i^\top E_q(\boldsymbol{\theta}_\beta)\right)\right)\Phi_\eta^\top
\end{aligned}$$

$$\begin{aligned}
\sigma_\epsilon^2 \mid \textsf{rest} &\sim \mathrm{IG}\left(\frac{1+NL}{2},\ \frac{1}{2}E_q\left(\|\widetilde{\mathbf{y}}_i - \mathbf{x}_i^\top \boldsymbol{\alpha}\widetilde{\Phi} - \mathbf{x}_i^\top \boldsymbol{\theta}_\beta - \boldsymbol{\theta}_{\eta_i}\Phi_\eta\|_2^2\right) + \frac{1}{E_q(a_\epsilon)}\right) \\
a_\epsilon \mid \textsf{rest} &\sim \mathrm{IG}\left(1,\ \frac{1}{A^2} + \frac{1}{E_q(\sigma_\epsilon^2)}\right) \\
\sigma_\eta^2 \mid \textsf{rest} &\sim \mathrm{IG}\left(\frac{1+NL_\eta}{2},\ \frac{\sum_{i=1}^N E_q(\|\theta_{\eta_i}\|_2^2)}{2} + \frac{1}{E_q(a_\eta)}\right) \\
a_\eta \mid \textsf{rest} &\sim \mathrm{IG}\left(1,\ \frac{1}{A^2} + \frac{1}{E_q(\sigma_\eta^2)}\right) \\
\sigma_\beta^2 \mid \textsf{rest} &\sim \mathrm{IG}\left(\frac{1+(J+1)L}{2},\ \frac{\sum_{j=0}^J E_q(\|\theta_{\beta_j}\|_2^2)}{2} + \frac{1}{E_q(a_\beta)}\right) \\
a_\beta \mid \textsf{rest} &\sim \mathrm{IG}\left(1,\ \frac{1}{A^2} + \frac{1}{E_q(\sigma_\beta^2)}\right) \\
\sigma_\alpha^2 \mid \textsf{rest} &\sim \mathrm{IG}\left(\frac{1+J+1}{2},\ \frac{\sum_{j=0}^J E_q(\alpha_j^2)}{2} + \frac{1}{E_q(a_\alpha)}\right) \\
a_\alpha \mid \textsf{rest} &\sim \mathrm{IG}\left(1,\ \frac{1}{A^2} + \frac{1}{E_q(\sigma_\alpha^2)}\right)
\end{aligned}$$
